# Effects of Specific RAMP Protocol Phase on Change of Direction Speed of Police Students

**DOI:** 10.3390/jfmk9040194

**Published:** 2024-10-13

**Authors:** Filip Kukić, Nemanja Zlojutro, Darko Paspalj, Senka Bajić, Saša Kovačević, Lazar Vulin, Nenad Rađević, Nenad Koropanovski

**Affiliations:** 1 Biomechanics Laboratory, Faculty of Physical Education and Sports, University of Banja Luka, 78000 Banja Luka, Bosnia and Herzegovina; nemanja.zlojutro@ffvs.unibl.org; 2 Faculty of Security Sciences, University of Banja Luka, 78000 Banja Luka, Bosnia and Herzegovina; darko.paspalj@fbn.unibl.org (D.P.); sasa.kovacevic@fbn.unibl.org (S.K.); lazar.vulin@fbn.unibl.org (L.V.); nenad.radjevic@fbn.unibl.org (N.R.); 3 Faculty of Technical Sciences, University of Novi Sad, 21102 Novi Sad, Serbia; senka.bajic@uns.ac.rs; 4 Umbra Lab, 21000 Novi Sad, Serbia; 5 Department of Criminalistics, University of Criminal Investigation and Police Studies, 11000 Belgrade, Serbia; nenad.koropanovski@kpu.edu.rs

**Keywords:** tactical athletes, law enforcement, tactical strength and conditioning, mobility, warm-up

## Abstract

**Background:** This study assessed the effects of two distinct RAMP (Raise, Activate, Mobilize, Potentiate) protocols, mobility-focused and reactiveness-focused, on change of direction speed in police students (i.e., tactical athletes). **Methods:** A longitudinal design with two experimental and one control group was employed. The study sample consisted of 39 police students (aged 19.2 ± 0.2 yrs) who were randomly allocated into three equal groups of 13 participants (7 females and 6 males). Experimental groups were labeled as the mobility group or reactiveness group based on the type of RAMP protocol they performed. During the tactical physical education classes, the mobility group performed four complex mobility exercises, while the reactiveness group performed four exercises for trunk reactiveness. After the specific warm-up, both groups continued with syllabus activities. The control group performed only regular activities based on the study syllabus. All participants performed the Illinois Agility test unloaded (IAT) and loaded (10 kg vest [IATL]) and Functional Movement Screening (FMS) before and after 8 weeks of the applied protocols. **Results:** In general, improvements were observed across all participants in the IAT (*p* < 0.001), IATL (*p* < 0.001), and FMS (*p* < 0.001). The mobility protocol had a more substantial impact compared to the reactiveness protocol on the IAT (d = 0.55 vs. d = 0.40), IATL (d = 0.44 vs. d = 0.38), and FMS (d = 0.88 vs. d = −0.42). Additionally, the control group, which did not follow either RAMP protocol, did not show significant improvements. **Conclusions:** These results underscore the importance of incorporating targeted mobility training in the limited time available for strength and conditioning programs, as it improves occupationally relevant movement qualities such as change of direction speed ability. Prioritizing mobility training in young tactical athletes may offer broader benefits compared to reactiveness training.

## 1. Introduction

Law enforcement officers often dedicate a significant portion of their work shifts seated in a patrol car or at a desk, completing reports. Conversely, this predominantly sedentary occupation can be interrupted with episodes of high-intensity activity [1,2]. These rigorous tasks may encompass executing forced entry for search warrants, pursuits and apprehensions of suspects, lifting heavy objects, engaging in close-quarters hand-to-hand combat, and swiftly maneuvering on foot to respond to situations [3]. In order to effectively perform these tasks, officers must maintain sufficient levels of physical fitness [4,5]. The transitions from sedentary periods to bursts of high-intensity work can frequently occur unexpectedly, limiting officers’ ability to adequately prepare themselves [2,6]. The heightened risk of injury, illness, and mortality is often linked to these unpredictable shifts in behavior. Therefore, it is imperative that officers attain and maintain physical fitness to protect their overall health and perform their job duties safely and effectively throughout their careers [1,7].

Studies suggest that recruits who enter law enforcement with higher physical fitness are better off at graduation [8,9,10] and better prepared to handle the demanding and unpredictable nature of the job [11,12]. Recruit fitness is crucial for enhancing operational readiness, reducing injuries, promoting long-term health, and ensuring high standards of performance within law enforcement agencies [6,13]. The most recent study by Gonzales et al. [14] found that recruits from 2019 had worse indicators of health and fitness compared to recruits from 2009, 2006, and 2003. This emphasizes the need for well-planned and executed recruit training that will increase their health and performance potential. According to Shusko et al. [15], recruits who struggled to complete a higher number of push-ups in 60 s and recorded slower times for a 2.4 km run before starting the academy were at a greater risk of leaving the academy. Furthermore, Lockie et al. [16] showed that maximal running performance was correlated with improved results in occupational tests for law enforcement recruits. These findings suggest that strength and conditioning programs for recruits must be designed to meet the physical fitness status of all recruits rather than using a one-size-fits-all approach.

Targeted strength and conditioning programs to enhance or sustain the fitness of police students and law enforcement officers were shown to be an effective strategy [17,18,19,20]. Cocke et al. [17] investigated the effects of two different physical training programs and reported that both programs could be used effectively to improve the fitness of tactical athletes. Cvorovic et al. [18] reported that a 12-week physical training program utilizing mesocycles and midpoint assessments can effectively enhance the fitness of participants even amidst their rigorous academy commitments. Furthermore, Massuça et al. [21] analyzed changes in the physical fitness of Portuguese police cadets over four years of the academy and found that their fitness improved even with the two physical education and sports sessions per week throughout the academy. In addition, Stojkovic et al. [20] found that a 10-week training program for overweight and obese police officers could be effective in reducing body anthropometric characteristics as well as in improving physical fitness measures. Therefore, it is evident that tailored strength and conditioning programs are an effective approach to improving the fitness of cadets and police officers.

While training, in general, has been evaluated, studies investigating the potential of different training phases (e.g., warm-up and cool-down phase) to influence fitness measures relevant to police occupation are scarce. Warm-up is of particular interest as it could be defined as a protocol consisting of a few subphases, each of which has a particular role. The often-used warm-up protocol that includes the Raise, Activate, Mobilize, and Potentiate phases (i.e., RAMP protocol) provides an opportunity to engineer the warm-up phase [22] to be repeatedly performed to provide a targeted effect on certain abilities. This approach allows strength and conditioning coaches to impact abilities that are not the main target of the training program. For instance, when the main aim of the training program is strength improvement, the warm-up phase could be used to work on mobility, stability, and/or agility. However, this needs to be tailored and implemented with precision, considering the duration of the training session and the main goal.

Core stability exercises are typically included in most RAMP protocols to enhance the spine stability and the trunk’s role in arm-leg connection. Mobility, another typical part of RAMP, focuses on improving the performance of prime movers while keeping the correct posture (i.e., stable posture). Although mobility and stability individually were shown to enhance change of direction speed performance [23,24], which one could be more potent in given circumstances has not been sufficiently investigated. Therefore, the aim of this study was to investigate the effects of two different RAMP protocols, one with a focus on stability and one with a focus on mobility, on the change of direction speed performance of young tactical athletes. It was hypothesized that (1) both protocols would have a positive impact on change of direction speed and (2) that mobility training would be more potent.

## 2. Materials and Methods

### 2.1. Experimental Approach to the Problem

This study used a longitudinal design with two experimental and one control group. Experimental groups were labeled as mobility groups and reactiveness groups based on the type of RAMP protocol they performed, while the control group performed regular activities from the study syllabus. During the tactical physical education classes, a specific RAMP protocol was designed for the mobility group, involving four complex mobility exercises, and for the reactiveness group, involving four exercises for trunk reactiveness. The control group performed a warm-up consisting of run and jog variations, calisthenics, and active and ballistic stretching. All participants performed the Illinois Agility (IAT) test and Functional Movement Screening test before baseline and after 8 weeks of applied protocols (post-treatment). The IAT was performed without and with the 10 kg loading vest (IATL).

### 2.2. Participants

This study included 39 (female = 21, male = 18) second-year students from the Faculty of Security Sciences, University of Banja Luka, Bosnia and Herzegovina. The main characteristics of the male participants were as follows: age = 19.2 ± 0.2 yrs, height = 181.5 ± 4.8 cm, weight = 78.1 ± 6.2 kg, and BMI = 23.6 ± 3.1 kg/m^2^. The main characteristics of female participants were as follows: age = 19.1 ± 0.2 yrs, height = 170.2 ± 5 cm, weight = 63.2 ± 4.5 kg, and BMI = 22.3 ± 3.3 kg/m^2^. The sample was randomly divided into three equal groups of 13 participants: control (female = 7, male = 6), mobility (female = 7, male = 6), and reactiveness (female = 7, male = 6) groups. All participants had regular specialized physical education, including self-defense and use of force techniques, as well as strength and conditioning classes. They passed the recruitment process and enrolled in September of 2022. The study was conducted in September and October of 2023 with students who completed the first year and started the second. At the beginning of the semester, students of the Faculty of Security Sciences were randomly divided into a few equal groups by size, sex distribution, and physical fitness level. Of these groups, three were used for the purposes of this study. The sample of this study was relatively homogenous due to the following: First, the recruitment process for the Faculty of Security Sciences includes physical fitness assessment and age limit. Second, we used second-year students (i.e., students further selected by the study process). Third, the study groups were formed randomly; thus, there was no significant between-group difference at baseline in age, physical fitness, and anthropometric characteristics. All participants were free of illnesses and injuries. The ethics committee provided the ethical approval for this study (No. 11/1.694/24). The study was conducted in accordance with the Helsinki Declaration [25].

### 2.3. Procedures

#### 2.3.1. Warm-Up Protocols

The control group used a non-specific warm-up protocol where participants went through phases of the RAMP protocol with the aim of preparing for the tasks of the tactical physical education classes. There was no specific highlight on any of the RAMP phases. Participants would perform 1–2 min of jogging, running, jumping jacks, etc.; 1–2 min of muscle activation exercises such as inchworm, crab walk, and plank variations; and 1–2 min of mobility exercises such as deep squat, lunge, and side lunge. The potentiation phase was connected to the main part of the class, following the demonstration of tasks.

The mobility group performed the same until the mobility phase, which was structured and lasted longer (about 5 min). The mobility phase included 4 exercises (Table 1) performed one after another with a short rest between exercises. Two rounds of six repetitions were performed. The focus was on the quality of movement.

The reactiveness group performed the structured activation phase and other phases in the same way as the control group. The activation phase included 6 exercises (Table 1) performed in work at stations. Participants performed 2 sets at one station and then changed the station. Those from station 1 transferred to station 2, from station 2 to station 3, and so on. Exercises were performed in pairs so that one participant was randomly applying lateral pressure to another participant’s hands extended forward with palms connected in the first three exercises (i.e., stations) and hands extended overhead in the fourth. The participant who was resisting the pressure was instructed to counter-resist as quickly as possible, not allowing large movements of hands and the body.

#### 2.3.2. Performance Assessment

Change of direction speed was assessed using the Illinois Agility Test. This test has often been used to assess the change of direction speed ability of tactical athletes [26,27]. The test setup and performance were conducted following the procedure reported in previous studies [26]. On the 5 × 10 m square, baseline and top line ends (5 m) were marked by cones, which also marked left and right line ends (10 m). In the middle of the square, 4 cones were positioned to mark the middle of the baseline and topline, and three equal sections in between (i.e., 2 cones at 3.3 m distance). Starting from the left end of the baseline, participants were instructed to run straight as fast as possible, make a right-hand turn around the top-line left-end cone, and run toward the middle cone on the baseline. There, participants made a left-hand turn and ran towards the topline as fast as possible, avoiding the two cones positioned in the middle of the square. At the topline middle cone, they performed a right-hand U-turn and ran, avoiding the cones towards the baseline, where they performed another left-hand side turn and sprinted towards the cone at the right top-line end. There, participants performed another right-hand side turn and sprinted to cross the baseline as fast as possible. Once the baseline was crossed, the test ended. The timing gate system HISENSE (Microgate, Bolzano, Italy) was used to measure the time needed to complete the test. The reliability and validity of this test have been reported elsewhere [28].

The Functional Movement Screening (FMS) test was used to assess participants’ mobility [29,30]. In short, the test consists of 7 movement patterns: deep squat, hurdle step, incline luge, shoulder mobility, active straight leg rise, trunk stability push-up, and rotatory stability. Each of these patterns was scored quantitively from 0 to 3. Each score had a qualitative description, with 0 being the lowest score attributed to the inability to perform the pattern due to pain and 3 being the highest score when the movement pattern could be performed without any compensations. The test was designed to assess one’s overall movement ability, including the ability of the trunk to stabilize the body and transfer forces so legs and arms could perform. The validity and reliability of this test have been reported elsewhere [31].

### 2.4. Statistical Analyses

Analyses were performed using the JASP statistical software (version 0.18.3, Amsterdam, The Netherlands). Descriptive data are shown for mean and standard deviation. The normality of data distribution was checked using the Shapiro–Wilk test and all variables were normally distributed. Thus, parametric tests were used to investigate the differences in results obtained at post-treatment against baseline measurements (i.e., treatment effects). A repeated measure ANOVA was used to investigate the effects of the specific RAMP protocol (treatment) and interaction treatment*group on outcome measures. The significance was set at *p* < 0.05. Chen’s effect sized (d) was calculated and interpreted as trivial < 0.2, small = 0.2–0.5, moderate = 0.5–0.8, large = 0.8–1.2, and very large > 1.2 [32]. However, using the GPower (v 3.1.9.4, Franz Faul, University of Kiel, Kiel, Germany), we calculated the minimum effect size of 0.53 for the given sample size and α.

## 3. Results

Descriptive stats and differences on a general level are shown in Table 2. Significant changes occurred in IAT (*t*_1_ = 7.33, *p* < 0.001, d = 0.40), IATL (*t*_1_ = 6.59, *p* < 0.001, d = 0.38), and FMS (*t*_1_ = −4.98, *p* < 0.001, d = −0.66).

When considering specific training groups, significant improvements in the IAT and IATL occurred in groups that applied mobility and reactiveness training but not in the control group (Table 3). Note that there was no between-group difference in baseline values of the IAT and IATL. While some differences in the FMS could be observed in the control and reactiveness groups, significant changes with large effect sizes occurred only in the mobility training group.

**Figure 1:**
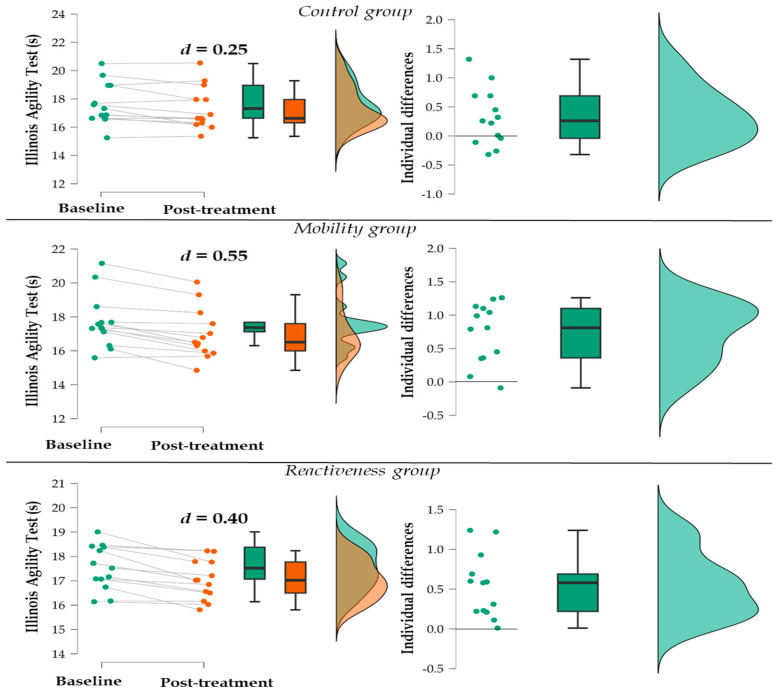
Distribution of participants at baseline and post-treatment and individual differences in the Illinois Agility Test for each group. Note: d–Cohen’s effect size.

Considering individual results for each group, four participants from the control group, one from the mobility group, and none from the reactiveness group ran the IAT slower at the post-treatment assessment (Figure 1). Furthermore, one from the control group, six from the mobility group, and two from the reactiveness group completed the IAT more than 1 s earlier (i.e., run faster) at the post-treatment compared to baseline.

**Figure 2:**
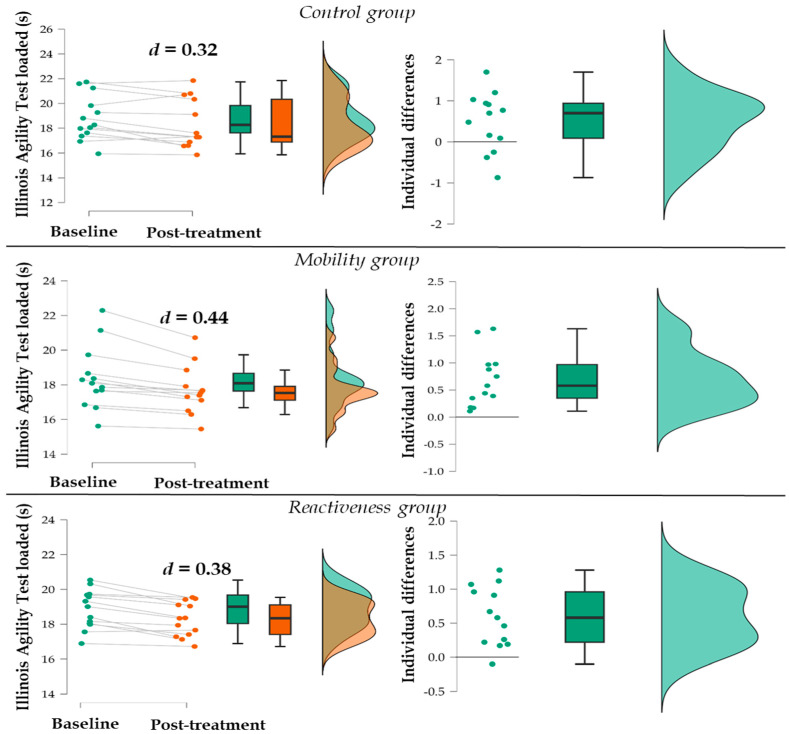
Distribution of participants at baseline and post-treatment and individual differences in the Illinois Agility Test Loaded for each group.

In IATL, three participants from the control and one from the reactiveness group ran slower, while in the mobility group, all participants ran faster at the post-treatment test compared to the baseline (Figure 2). Improvements greater than 1 s occurred in three, two, and three participants in the control, mobility, and reactiveness groups, respectively.

**Figure 3:**
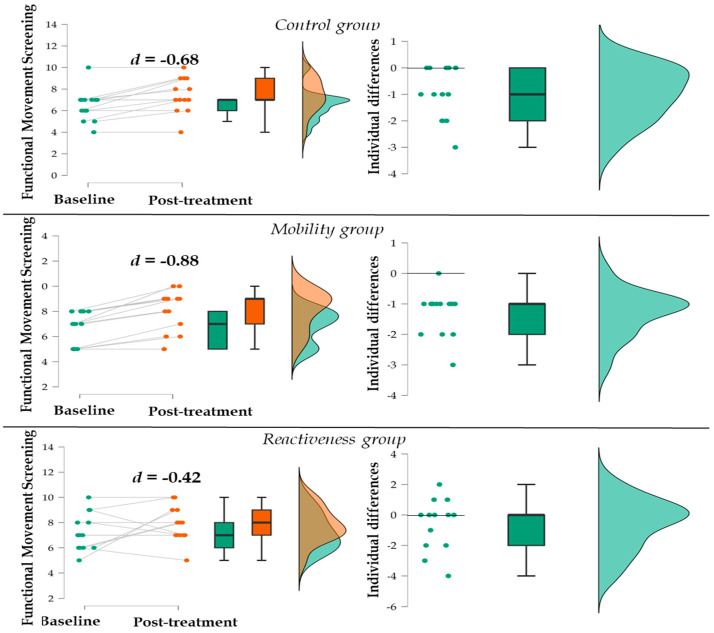
Distribution of participants at baseline and post-treatment and individual differences in the FMS for each group.

Considering the FMS, five (38.4%) participants in the control, one (7.7%) in the mobility, and five (38.4%) in the reactiveness group obtained the same result at the post-treatment compared to baseline (Figure 3). Note that three participants from the reactiveness group performed better at baseline compared to post-treatment (i.e., worsened after the treatment).

## 4. Discussion

This study investigated the effects of two different RAMP protocols on the change of direction speed performance and mobility in police students. The main findings suggest that both the reactiveness and mobility group improved their change of direction speed performance and FMS score. However, greater improvements occurred in the mobility group. It is important to note that improvements could also be observed when all participants were analyzed together, which could mask the effects in individual groups. Thus, the hypotheses of this study were true, providing a clear understanding of the utilization of specific RAMP protocols in the optimization of the performance in this population that typically has limited time for strength and conditioning programs.

Considering the frequency and weekly schedule of the specialized physical education classes, one may argue that improvements on a general level are expected. However, our results showed that the control group did not improve significantly in the two assessed movement abilities. Thus, it is probable that the specialized physical education program alone is not sufficient to improve change of direction speed, ability, and mobility. This means that through their overall educational program, police students exit their education and enroll as officers with small or no improvement in two occupationally very important movement qualities. The change of direction speed and mobility are important not only for occupational performance but are also protective against injury occurrence [33,34,35]. Improvement of these two qualities requires carefully engineered training programs so tactical athletes pay attention to it yet do not spend too much time in their time-limited classes.

Implementation of a specific warm-up protocol significantly improved change of direction speed performance without and with the load like that carried by police officers. The mobility protocol had the greatest systematic effect as all participants from the mobility group improved their IAT performance in the unloaded and loaded. The reactiveness protocol also showed potential for improvement in both IAT performances, but the effect size was smaller in the unloaded IAT compared to the mobility group. Mobility refers to one’s ability to perform movement in a stable and coordinated manner [22,23], all of which contribute to the effectiveness of task performance. Furthermore, mobility training improves body awareness and connection with the ground, enabling the activation of prime movers and essentially supporting and stabilizing muscles more effectively [22]. Reactiveness training, on the other hand, focuses on enhancing the ability to maintain or regain position or movement trajectory after sudden disturbances [24,36]. Therefore, emphasis on trunk reactiveness may increase trunk stability and rigidness [24,37], resulting in a small improvement in change of direction speed.

Expectedly, the mobility protocol resulted in improved functional mobility, which was found to be related to reduced risk of injuries. This further emphasizes the practicality of the carefully drafted mobility section within the RAMP protocol (i.e., warm-up section) [22,24]. Based on the results, when training time and frequency are limited, prioritizing mobility over reactiveness in young, healthy tactical athletes may be advantageous due to its broader spectrum of benefits. In addition, improvements in mobility typically include enhancements in stabilization and reactiveness, whereas the reverse is not necessarily true. It is of note, however, that the context and sample also may play a role in the effects obtained. Namely, the sample of this study was relatively homogenous due to the selection process and comprised participants whose physical fitness was already at a good level. Considering that their specialized physical education classes included the use of force and self-defense activities, which have similarities with the reactiveness protocol, it may be that this lowered the participants’ adaptation to the reactiveness protocol. This strengthens the notion that the emphasis on mobility in a warm-up may be advantageous over trunk reactiveness if the aim is to improve mobility and the change of direction speed of tactical athletes.

### Limitations

The sample of each group could be extended to all other students from this study year. This would test the systematic applicability of this approach. The age span was narrow because the study included only police students from the university, who were selected (among other criteria) on their age as well. The age range could be extended by applying this approach at the agency level. The subsamples by sex could be bigger, which would provide the possibility of including sex as a factor in statistical analysis. However, considering the occupational demands and loads of policing jobs, it was reasonable to perform the statistics using the unified sample, too. Body composition was not checked at the end of an 8-week intervention. While we do understand that changes in body composition could be a contributing factor, it is less likely that participants improved solely based on changes in body composition as these were young and relatively fit participants. It is also less likely that only one group improved body composition more than the other two groups as none of the training groups performed training that could induce changes in body composition superior to other groups.

## 5. Conclusions

The findings of this study suggest that incorporating a mobility-focused RAMP protocol into the training of police students can lead to significant improvements in change of direction speed and mobility, both of which are critical for their occupational performance and injury prevention. Given the limited time available for strength and conditioning programs, prioritizing mobility exercises within warm-up routines could offer substantial benefits, including enhanced stabilization and reactiveness. These results indicate that implementing targeted mobility training could be a practical and effective strategy for optimizing the physical preparedness of tactical athletes, ensuring they are better equipped for the demands of their profession. In addition, it should be pointed out that reactiveness warm-up also resulted in some improvement, thus suggesting that the warm-up should not be reserved for mobility only. Rather, using the RAMP protocol allows for certain creativeness to avoid training monotony.

## Figures and Tables

**Table 1:** Exercises applied in mobility and reactiveness groups.

Mobility	Reactiveness
Overhead deep squat to deadlift position (5 kg plate); High plank to Spiderman lunge with rotation to crab walk position; Hand-supported deep side lunge; Lizard Walk.	Wide parallel stance with hands in front; Right-legged lunge position with hands in front; Left-legged lunge position with hands in front; Wide parallel stance with hands overhead.

**Table 2:** Descriptive statistics.

Variable	Test	Control		Mobility		Reactiveness	
		Mean	Std. Dev.	Mean	Std. Dev.	Mean	Std. Dev.
IAT (s) ***	Baseline	17.65	1.47	17.70	1.56	17.55	0.92
	Post-treatment	17.33	1.51	16.97	1.48	17.01	0.8
IATL (s) ***	Baseline	18.82	1.83	18.38	1.8	18.86	1.11
	Post-treatment	18.32	1.98	17.68	1.38	18.26	0.98
FMS (pts) ***	Baseline	6.46	1.45	6.77	1.3	7.23	1.48
	Post-treatment	7.46	1.61	8.08	1.61	7.85	1.41

Note: *** Significant at *p* < 0.001. IAT–Illinois Agility Test, IATL–Illinois Agility Test Loaded, FMS–Functional Movement Screening.

**Table 3:** Between-group differences at the baseline and within-group differences at the post-treatment.

Group-Treatment Interaction		IAT		IATL		FMS	
		Mean Diff.	Cohen’s d	Mean Diff.	Cohen’s d	Mean Diff.	Cohen’s d
Control Baseline	Mobility, Baseline	−0.05	Trivial	0.44	Small	−0.31	Small
	Reactiveness, Baseline	0.11	Trivial	−0.04	Trivial	−0.77	Moderate
	Control, Post-treatment	0.33	Small	0.5	Small	−1	Moderate
Mobility Baseline	Reactiveness, Baseline	0.15	Trivial	−0.49	Small	−0.46	Small
	Mobility, Post-treatment	0.73 ***	Moderate	0.69 **	Small	−1.31 **	Large
	Reactiveness, Post-treatment	0.69	Moderate	0.11	Trivial	−0.62	Moderate
Reactiveness Baseline	Reactiveness, Post-treatment	0.53 **	Small	0.6 **	Small	−0.31	Small

Note: ** *p* < 0.01, *** *p* < 0.001. IAT–Illinois Agility Test, IATL–Illinois Agility Test Loaded, FMS–Functional Movement Screening.

## Data Availability

Data is available upon request: filip.kukic@ffvs.unibl.org.
